# Gardener's Nightmare: A Rare Case of Myroides-Induced Septic Shock

**DOI:** 10.7759/cureus.12235

**Published:** 2020-12-23

**Authors:** Rucci Marcus Foo, Sushant M Nanavati, Anish Samuel, Ruth Lamm, Shivanck Upadhyay

**Affiliations:** 1 Department of Internal Medicine, Beth Israel Deaconess Medical Center, Harvard Medical School, Boston, USA; 2 Department of Pulmonary and Critical Care Medicine, St. Joseph's Regional Medical Center, Paterson, USA; 3 Department of Critical Care, St. Joseph's Regional Medical Center, Paterson, USA

**Keywords:** myroides, septic shock, immunosuppression, contaminated soil, multi-drug resistant bacteria

## Abstract

Myroides species, an uncommon clinical isolate, generally found in contaminated sources of environment, is an emerging source of infections, especially amongst immunocompromised patients. Though only 54 cases have been reported to our knowledge, the emergence of pan-resistance to antibiotics remains a concern that may burden healthcare and require awareness. We present the case of an elderly female who despite being home-bound, without any environmental exposure, contracted Myroides septicemia that progressed to septic shock and showed resistance to usual empiric antibiotics. In our case, the patient was exposed to contaminated soil via her family and was successfully treated with carbapenem. The case provides awareness amongst clinicians to suspect this emerging yet threatening infection within immunocompromised patients.

## Introduction

Myroides spp. are an extremely rare cause of bacteremia in humans. This genus of bacteria is of the family Flavobacteriaceae and includes aerobic environmental bacteria that dwell in soil and water. Few cases have been identified in the medical literature and even fewer cases have led to florid bacteremia. We present the 55th published case of Myroides in humans, the majority of which cause soft tissue infections [1]. Lack of obvious site of entry in a home-bound patient as well as documented multidrug antibiotic resistance suggest that Myroides is a formidable source of bacteremia in the immunocompromised in the context of intensive care unit admissions.

## Case presentation

An 87-year-old white woman presented to the emergency department for elevated blood sugar, difficulty swallowing, and lethargy. She had a medical history of diabetes mellitus, hypertension, gastroesophageal reflux disease, and dyslipidemia. The patient lived with his son (caretaker) and has not left her home for over four years and house calls are made by her primary care physician and home health nurse. On admission, her temperature was 36.7°C (98.1°F), heart rate 111 beats per minute, respiration rate 16 breaths per minute, saturating 96% on room air, and blood pressure 76/48 mmHg. Physical exam showed a frail, older woman with altered mental status who was awake and alert without the ability to recall the place, time, and events. She complained of right-foot and leg pain secondary to stasis ulcers from bilateral lower extremity edema and eschars. Laboratory tests showed an elevated white blood cell count of 11.5 x 109/L with a band cell proportion of 46%, Hemoglobin 11.8 g/dL, Hematocrit 38.9%. Her blood sugar was 221 mg/dL, troponins 0.052 ng/mL, blood urea nitrogen 42 mg/dL, creatinine 2.06 mg/dL, potassium 6 meq/L, alkaline phosphatase 179 units/L, phosphate 6.3 mg/dL, and lactic acid 7.6 mEq/L. Procalcitonin was 14.99. Urinalysis demonstrated large leukocyte esterase and bacteria. The patient was admitted to the medical intensive care unit for septic shock, acute encephalopathy, and acute kidney injury.

Despite being started on appropriate intravenous fluid resuscitation and empiric broad-spectrum antibiotics, vancomycin and piperacillin-tazobactam, the patient continued to remain hypotensive and required initiation on a continuous vasopressor (norepinephrine) infusion to achieve a mean arterial pressure (MAP) more than 65 mm Hg. After two days, her blood cultures grew gram-negative bacteria that subsequently were identified as Myroides species. Once the cultures revealed Myroides that is typically found in contaminated soil, the patient's son revealed his hobby of performing gardening in the yard. Based on the sensitivities of the culture, the patient was started on meropenem. Subsequently, the patient was weaned off the vasopressor since her MAP remained above 65 mm Hg; her mental status and appetite also improved. The patient was moved to the medical floor after her appetite and mental status improved. The patient was scheduled for discharge when suddenly she was noted to be unresponsive; the patient's family opted for comfort measures. Later, the patient expired in the hospital.

## Discussion

This presentation of Myroides is of significance, not only because of its rarity as an infection but because of an unclear route of entry into the body. Myroides most commonly dwells in soil and water and rarely becomes pathogenic in humans [2]. In the context of a home-bound patient, this becomes even more perplexing. It is worth noting that some researchers have identified an increase in Myroides identification over the last decade [3]. While the patient’s chronic ulcerations and eschars on the right lower extremity seemed like promising sites of entry, a culture of the site revealed no presence of Myroides. Other cases identified worldwide reported exposure to soil and water-filled environments, e.g. walking through melted snow, animal bites, and nature walks [1,2]. In the context of our patient, no clear source was identified. Wound care was a priority for this patient and most of her home medications were for the treatment of her chronic leg ulcers. The patient’s relative immunocompromised status due to her diabetes mellitus and advanced age may have contributed to the ability of the bacteria to enter the bloodstream.

Multidrug resistance in Myroides can be attributed to several factors. Mammeri et al and other groups have identified chromosomally encoded β-lactamases and other metalloenzymes that are capable of hydrolyzing carbapenems as well as other β-lactam antibiotics [4]. According to Hu et al, 16S ribosomal RNA analysis can identify strains but fails to demonstrate mechanisms of antibiotic resistance. However, similar strains of the genus employ similar mechanisms of resistance [5]. This case demonstrates the importance of extended susceptibility testing in suspected Myroides infection.

## Conclusions

Myroides is a formidable genus of bacteria associated with opportunistic infections in immunocompromised patients. In critically ill patients, a thorough history of exposure should be obtained as well as extended susceptibility testing should be performed if an infection is suspected.
